# From epigenetic mark detection to rational design of epigenetic editing strategies

**DOI:** 10.1016/j.omta.2026.201831

**Published:** 2026-08-04

**Authors:** Ali Faiq, Sibtain Haider, Claudio Mussolino

**Affiliations:** 1 Institute for Transfusion Medicine and Gene Therapy, Medical Center—University of Freiburg, 79106 Freiburg, Germany; 2 Center for Chronic Immunodeficiency (CCI), Medical Center—University of Freiburg, 79106 Freiburg, Germany; 3 Faculty of Medicine, University of Freiburg, 79106 Freiburg, Germany

**Keywords:** epigenetic marks, programmable epigenetic modifiers, epigenetic mapping, CRISPR dCas9, TALEs

## Abstract

Although cells within an organism share nearly identical genomes, their transcriptional programs differ markedly due to reversible chemical modifications known as epigenetic marks. These marks, including DNA methylation and histone modifications, regulate gene expression without altering DNA sequence and play a central role in development and disease. While epigenetic drugs such as DNA methyltransferase inhibitors have shown clinical benefit, their genome-wide activity often results in off-target toxicity limiting broader therapeutic applications. This has driven the development of locus-specific epigenetic editing strategies. Programmable epigenetic modifiers (PEMs) combine customizable DNA-binding platforms, such as CRISPR-dCas systems, transcription activator-like effectors (TALEs), or zinc fingers, with epigenetic effector domains to precisely install or remove regulatory marks at defined genomic loci. Because effective editing depends on the pre-existing epigenetic landscape, detection and characterization of target-site epigenetic states is a prerequisite for rational editor design, increasingly aided by machine-learning models that predict editing outcomes. In this review, we summarize current technologies for epigenetic mark detection and discuss the transition from global pharmacological approaches to programmable, modular editing systems that enable spatial and temporal control of gene regulation. We further address heritability and delivery constraints. Reversible, site-specific epigenetic editing represents a promising therapeutic paradigm for cancer, genetic disorders, and regenerative medicine.

## Graphical abstract

**Figure:**
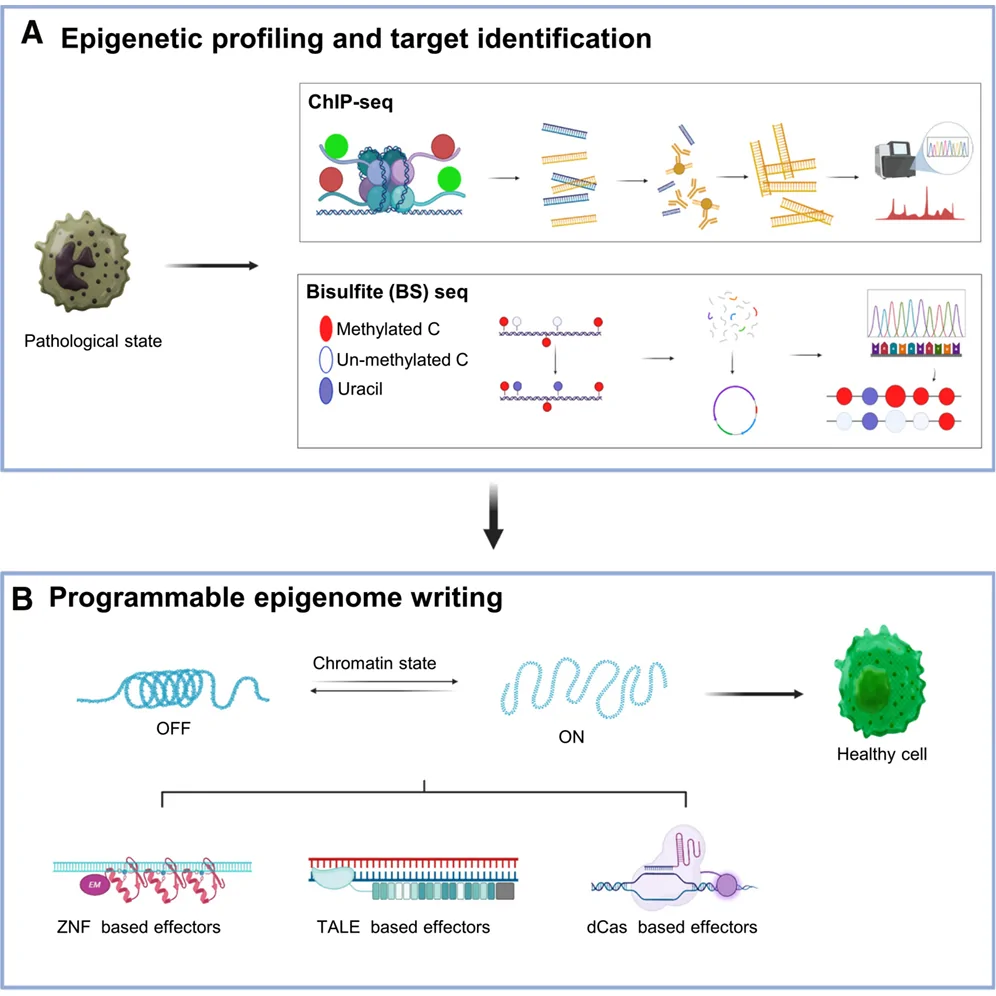

## Teaser

Faiq and colleagues examine how epigenetic mark detection can guide the rational design of programmable epigenetic editing strategies. They highlight advances in locus-specific targeting and effector selection, positioning reversible epigenome reprogramming as a promising therapeutic approach for monogenic and complex multifactorial diseases.

## Introduction

**Figure 1:**
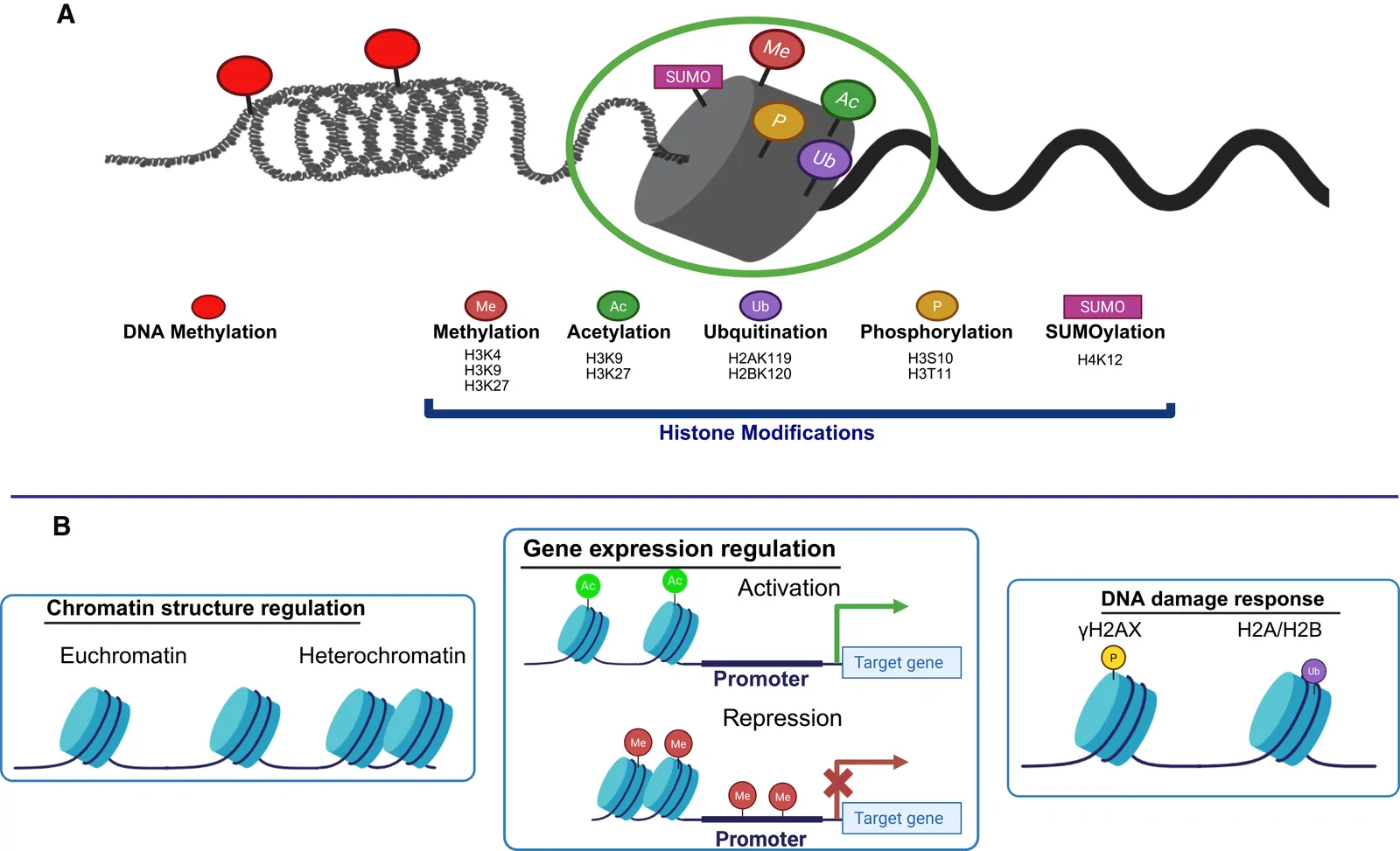
Major histone post-translational modifications and their functional roles (A) Overview of major histone post-translational modifications (PTMs), including methylation, acetylation, ubiquitination, phosphorylation, and SUMOylation, at selected histone residues. DNA methylation is also indicated. (B) Schematic representation of the roles of histone PTMs in chromatin organization, transcriptional regulation, and the DNA damage response. (Created with BioRender).

The concept of epigenetic regulation has evolved from Conrad Waddington’s predictive 1957 metaphor of developmental landscapes to become a cornerstone of modern molecular biology and therapeutic medicine.^1^ Epigenetic modifications, including DNA methylation, and histone post-translational modifications (Figure 1; Table 1), drive chromatin remodeling, and orchestrate precise spatiotemporal control of gene expression programs. These in turn define cellular identity and function,^2^^,^^3^ are both heritable and reversible, enabling dynamic responses to developmental cues, environmental stimuli, and pathological conditions while maintaining genomic stability.^4^^,^^5^

**Table 1:** Histone-modifying enzymes, target sites, functions, and disease associations

Name	Enzyme type	Enzyme name	Target site in histone	Function	Disease association
HDACs	class I	HDAC1	H3K9ac, H4K16ac	transcriptional repression	inflammatory diseases, cancer
		HDAC2	H3K9ac, H4K16ac	transcriptional repression, memory function	Alzheimer’s disease, cancer
		HDAC3	H3K9ac	transcriptional repression, immune regulation	inflammatory diseases
	class IIa	HDAC4	H3/H4 acetyl-lysines	cell differentiation	atherosclerosis, cardiac calcification
		HDAC5	H3/H4 acetyl-lysines	cardiac remodeling, gene expression	cardiac diseases
	class Iib	HDAC6	H4K12ac	protein degradation	neurodegeneration, cancer
	class IV	HDAC11	H3K9ac, H4K16ac	immune regulation	–
HAT	p300/CBP	p300	H3K27ac, H3K18ac, H4K12ac	transcriptional activation	neurodegeneration, cancer
	MYST	Tip60	H4K16ac, H2AK5ac	cognition, DNA repair, apoptosis	cognitive impairment
	GNAT	KAT2A/GCN5	H3K9ac, H3K14ac, H3K18ac	chromatin remodeling, transcriptional activation, cell proliferation	cancer (notably neuroblastoma), cardiomyopathy, ferroptosis, possibly neuroinflammatory disease
HMT		EHMT2 /G9a	H3K9	H3K9me2, represses gene expression	Prader-Willi syndrome, Angelman syndrome
		EZH2 (PRC2)	H3K27me3	gene silencing	cancer, neurodegeneration
		SETD2	H3K36me3	transcriptional elongation	cancer
		PRC2 (complex)	H3K27me3	silencing of tumor suppressors	cancer
HDM	LSD	LSD1/KDM1A	H3K4me1/2	transcriptional Regulation	cancer, neurodegenerative diseases
		LSD2/KDM1B	H3K4me1/2	Transcriptional Regulation	cancer
	JmjC family	KDM2A	H3K36me2	chromatin structure	cancer, neurodegeneration
		JMJD1C	H3K9me2	demethylation, macrophage regulation	atherosclerosis, inflammation
		JMJD2D	H3K9me3, H3K36me3	demethylation, genome stability	multiple cancers, drug resistance
		JMJD3 (KDM6B)	H3K27me3	demethylation, immune response	prostate cancer, lymphoma
		KDM7A	H3K9me2, H3K27me2	gene regulation	cancer, neurodegeneration

The therapeutic potential of targeting epigenetic mechanisms has been underscored through the clinical success of DNA methyltransferase inhibitors, such as 5-azacytidine and decitabine, alongside histone deacetylase inhibitors, including vorinostat and romidepsin.^6^^,^^7^ These agents have shown efficacy primarily in hematological malignancies, leading to their FDA approval and integration into standard oncological care.^8^ Their capacity to reverse widespread epigenetic silencing underlies clinical benefit in hematologic malignancies; however, this global mode of action inherently limits therapeutic precision and makes such approaches unsuitable for most non-malignant diseases that cannot tolerate broad epigenetic perturbation.^9^^,^^10^ Preclinical studies now demonstrate that locus-specific strategies for CpG demethylation or selective disruption of DNA methyltransferase 1 (DNMT1) can restore individual gene function without perturbing the broader epigenome. Although targeted approaches have not yet been clinically compared with approved epigenome-editing drugs, their mechanistic specificity and capacity to correct discrete pathogenic lesions highlight a clear conceptual advantage.^11^ These considerations have driven the development of next-generation programmable epigenetic modifiers (PEMs), designed to achieve precise, context-dependent chromatin modulation while minimizing systemic effects. Structurally, they are composed of DNA-binding domains (zinc finger proteins [ZFPs], transcription activator-like effectors [TALEs], and dCas) fused to either direct transcriptional modulators or epigenetic modifiers.^12^^,^^13^ Functionally they enable targeted gene regulation either through site-specific deposition or removal of epigenetic marks, or by recruiting endogenous cofactors that remodel chromatin architecture, modulate transcriptional complex assembly, and establish stable activation or silencing states that can be maintained as durable transcriptional memory.^14^^,^^15^

The modular nature of these tools enables the recruitment of diverse effector domains, including DNA methyltransferases (DNMTs), DNA demethylases (DDMs), histone acetyltransferases (HATs) and deacetylases (HDACs), and histone methyltransferases (HMTs) and demethylases (HDMs), offering comprehensive control over chromatin states.^16^^,^^17^ However, the optimal application of PEMs requires a comprehensive understanding of underlying chromatin landscapes and cellular contexts. Recent advances in epigenomic profiling technologies, including single-cell chromatin accessibility sequencing, multiplexed histone modification mapping, and direct nanopore methylation detection, have provided unprecedented insights into chromatin organization and dynamics.^18^^,^^19^ This review highlights the integration of epigenomic profiling with PEMs to create targeted treatments suited to different chromatin settings, while also briefly discussing machine-learning approaches to target design, the heritability of installed marks and delivery constraints relevant to therapy.

## Epigenetic detection technologies: Foundation for informed intervention

The pre-existing epigenetic landscape of a target locus is a major determinant of the outcome of programmable epigenetic editing. Therefore, comprehensive characterization of DNA methylation, chromatin accessibility, and histone modifications before intervention provides a rational framework for selecting the most appropriate epigenetic effector and maximizing editing outcome. Table 2 summarizes the principal technologies available for profiling these features and illustrates how they can guide the rational design of PEMs for targeted therapeutic applications.

**Table 2:** Classification of epigenetic analysis techniques by modality, application, strengths, and limitations

Category	Method	Application	Strengths	Weaknesses
PCR based	bisulfite PCR Sequencing^20^	locus-specific methylation	cost-effective single-base resolution detect all CpG sites	requires validated primers DNA degradation
	methylation-specific PCR(MSP)^21^	locus-specific methylation	cost-effective single-base resolution	low throughput limited CpG sites
	ChIP-PCR^22^	histone modifications	standard method cost-effective	only enrichment abundance no single-nucleotide resolution
Sequencing based	pyrosequencing^23^	locus-specific methylation	high quantitative accuracy cost-effective single-base resolution	requires validated primers DNA degradation
	whole genome bisulfite sequencing (WGBS)^24^	genome-wide methylation	most comprehensive single-base resolution	expensive needs bioinformatics high DNA input
	reduced representation bisulfite sequencing (RRBS)^25^	genome-wide methylation	covers gene regulatory CpG islands cost-effective single-base resolution	Covers only 1–3% genome misses intergenic regions
	MRE-Seq^26^	genome-wide methylation	cost-effective no harsh chemicals	low resolution site coverage depends on enzymes
	MeDIP^27^	genome-wide methylation	cost-effective sensitive in low CpG density	low resolution relies on antibody quality
	single-cell bisulfite sequencing^28^	single-cell methylation	single-cell resolution suitable for low-input samples	same as WGBS/RRBS bisulfite degradation complex
	SMRT sequencing^29^	long-read methylation detection	long-read native DNA sequencing detects multiple methylation types	high DNA input needed Mostly for bacteria
	nanopore sequencing	long-read methylation detection^30^	long-read sequencing native DNA broad species applicability	high DNA input Algorithm stability issues
	ChIP-seq^31^	histone modifications	most comprehensive genome-wide	high cost Needs quality antibody
Integrative	ChIP-BMS^32^	histone-DNA methylation	locus-specific methylation with histone context single-base resolution	requires high DNA input Cloning often needed
	BisChIP-seq^33^	histone-DNA methylation	genome-wide methylation with histone contextsingle-base resolution	high cost complex protocols bioinformatics required
	methyl-HiC^34^	3D chromatin & methylation	simultaneous chromatin and methylome profiling single-cell resolution	expensive complex needs Hi-C and bisulfite treatment

### DNA methylation

The accurate detection and quantification of DNA methylation patterns is a prerequisite for informed epigenetic editing strategies. Traditional bisulfite sequencing approaches, including whole-genome bisulfite sequencing (WGBS) and reduced representation bisulfite sequencing (RRBS), have provided foundational insights into methylation landscapes.^35^^,^^36^ However, these approaches are limited by chemical conversion artifacts, incomplete cytosine (C) to uracil (U) conversion rates, and inability to distinguish between 5-methylcytosine and 5-hydroxymethylcytosine modifications.^37^^,^^38^ Recent technological advances have addressed these limitations through the development of direct detection methods and enhanced computational approaches. Nanopore sequencing technologies have emerged as transformative platforms for direct DNA methylation analysis without chemical conversion requirements. Oxford Nanopore Technologies has pioneered the field and its proprietary direct methylation detection methodology demonstrates strong correlation with established pyrosequencing methods while extending analysis to repetitive genomic elements previously inaccessible to short-read approaches.^39^^,^^40^ This technology enables simultaneous detection of 5-methylcytosine and 5-hydroxymethylcytosine modifications across comprehensive genomic regions with single-molecule resolution.^41^ Single-cell bisulfite sequencing protocols have been optimized to enable methylation analysis from individual cells, revealing previously hidden cellular subpopulations and developmental trajectories.^42^ The integration of single-cell methylation data with transcriptomic and chromatin accessibility information provides comprehensive cellular state characterization that informs optimal intervention strategies.^43^^,^^44^

Some other technologies being used in DNA methylation studies include two-step qPCR-based methylation analysis in which bisulfite-converted DNA is first pre-amplified with methylation-independent primers, followed by nested methylation-specific qPCR to quantitatively assess CpG methylation at defined regulatory regions using ΔCt normalization.^45^ Additional approaches include enzymatic methods utilizing methylation-sensitive restriction enzymes, affinity-based enrichment strategies leveraging methyl-binding proteins or antibodies, and mass spectrometry-based platforms capable of high-resolution quantification.^46^ These approaches offer complementary strengths in sensitivity, specificity, and coverage, enhancing epigenetic research and clinical diagnostics.

### Histone modification profiling

Histone modifications play a key epigenetic role by regulating chromatin structure and influencing gene expression.^47^ The most commonly used technique for histone profiling is chromatin immunoprecipitation followed by sequencing (ChIP-seq), which defines the genomic locations of specific histone marks and reports on regulatory-element activity that guides editor choice.^31^^,^^48^ ChIP-seq usually requires high cell numbers, which makes it inefficient for human clinical samples, which are limited by low cell numbers and sample availability. To overcome this issue, more sophisticated techniques, such as CUT&Run and CUT&Tag have emerged, enabling profiling with as few as thousands or even single cells for the latter.^49^^,^^50^ However, these techniques require live cells, complicating their use in clinical settings due to sample handling constraints and batch effects.^50^^,^^51^ Alternatives like ChIPmentation allow for analysis of formaldehyde-fixed cells, offering more flexibility by enabling sample storage and synchronized processing.^52^ Building on this, a semiautomated, micro-scaled ChIP-seq protocol has been developed to further enhance throughput and reproducibility. This methodology is highly sensitive, enabling reliable profiling from as few as 10,000 cells while maintaining high speed and consistency.^53^^,^^54^

Because most of these assays require substantial input and profile one modification at a time, integrative methods (Table 2) that simultaneously resolve histone modifications and DNA methylation are increasingly valuable.^47^ To determine the most suitable editing strategy, the initial chromatin landscape at the target site must be analyzed by profiling DNA methylation and histone marks. These data help pinpoint the dominant regulatory barrier and thereby direct the choice of effector.

A growing emphasis in epigenetic detection is the move from bulk to single-cell and single-molecule resolution, which is critical because bulk profiling reports population averages and therefore masks cell-to-cell variation in chromatin state. Single-cell assays for chromatin accessibility (scATAC-seq) and single-cell histone-modification mapping (single-cell CUT&Tag and related scChIP approaches) directly resolve this heterogeneity, identifying distinct cellular subpopulations and the regulatory states present at a target locus across a mixed population.^55^ Complementing these, long-read sequencing platforms from Oxford Nanopore and Pacific Biosciences directly detect 5-methylcytosine on native DNA without bisulfite conversion and can phase methylation onto individual long molecules, enabling allele-specific methylation analysis and the resolution of methylation at repetitive regions that are inaccessible to short-read approaches.^56^^,^^57^ The integration of single-cell methylation data with transcriptomic and chromatin accessibility information provides comprehensive cellular state characterization that informs optimal intervention strategies.^43^^,^^44^ Resolving the target-site chromatin landscape at this level of detail provides a more precise foundation for selecting the appropriate effector and, after intervention, for characterizing the heterogeneity, allele specificity, and durability of the resulting epigenetic state.

### Chromatin context as a determinant of editing efficiency

Knowing how to read the chromatin landscape is only useful if that information guides the intervention, and the pre-existing chromatin state is itself a critical and often underappreciated determinant of editing outcome. The state at the target locus governs both the physical accessibility of the DNA-binding domain and the catalytic productivity of the recruited epigenetic effector. Because these effectors must act on a nucleosomal template, the local balance of activating and repressive marks can shift editing efficiency by orders of magnitude at otherwise identical target sequences. Systematic analyses of dCas9-based epigenome editors make this dependence explicit: when KRAB-, EZH2-, and DNMT3A-based editors were targeted across many endogenous loci, the efficiency and, in particular, the long-term heritability of silencing were strongly predicted by pre-existing chromatin features at the target site, with marks of active chromatin such as H3K27 acetylation associated with resistance to durable repression.^58^ This dependence operates at the most basic level of target engagement, as nucleosome occupancy can directly impede dCas9 binding, so that dense, compact chromatin occludes access while accessible regions permit efficient recruitment and effector activity.^59^ Mapping the chromatin landscape before intervention therefore allows the dominant regulatory barrier to be identified and the effector to be matched to it—for example, prioritizing demethylase or chromatin-opening activities at silenced, hypermethylated loci rather than assuming a single effector will perform uniformly across the genome.^60^ With this dependence in mind, the strategies available to modify the epigenome can be considered, ranging from global pharmacological agents that act without regard to locus to programmable editors designed to exploit or overcome the chromatin context described here.

## Strategies to modify the epigenome

### Epigenetic drugs and chemical modulators

**Figure 2:**
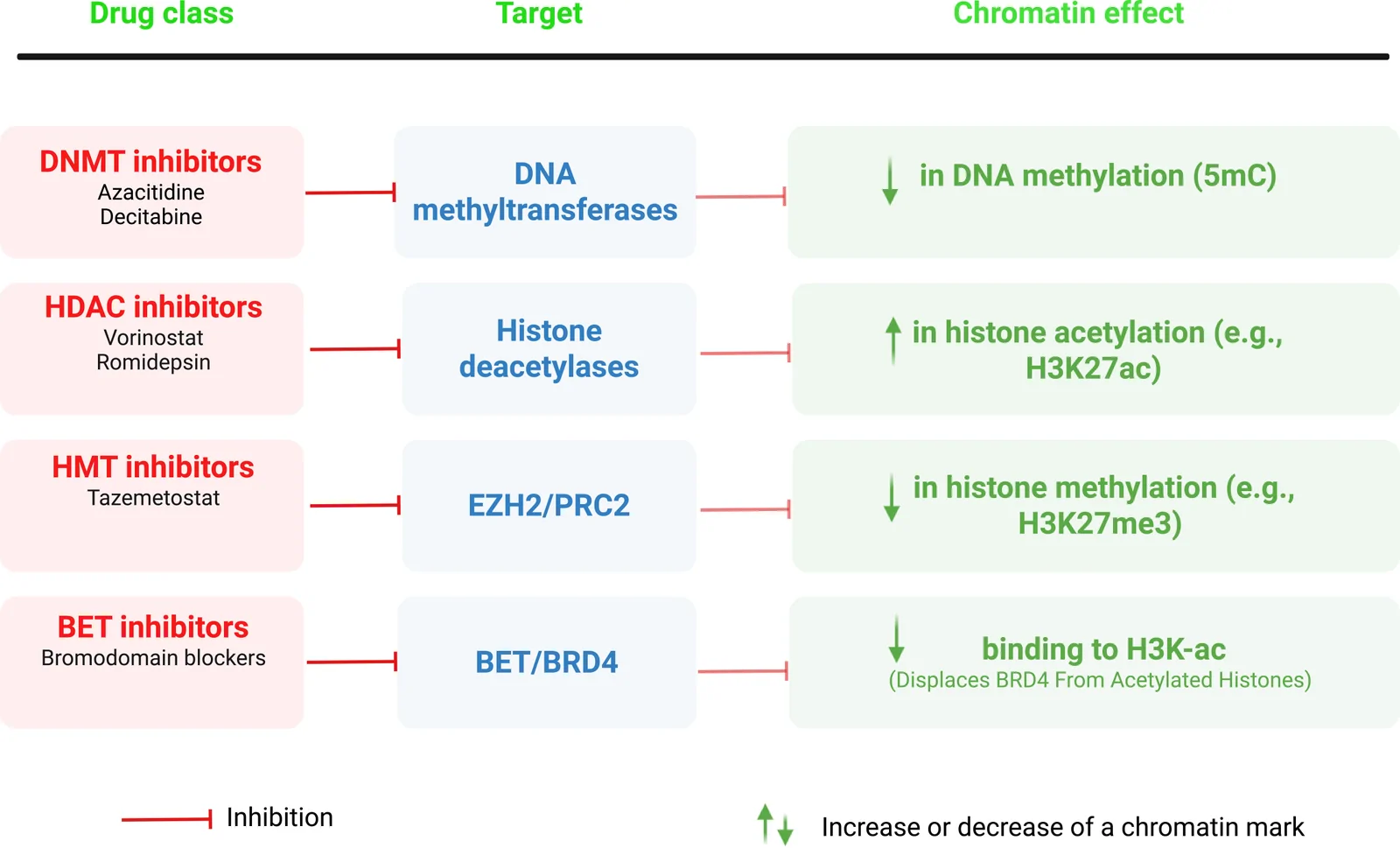
Major classes of small-molecule epigenetic drugs and their targets Schematic representation of selected classes of epigenetic drugs, including DNA methyltransferase (DNMT), histone deacetylase (HDAC), histone methyltransferase (HMT), and bromodomain and extra-terminal (BET) inhibitors, together with representative compounds, their molecular targets, and associated chromatin changes. Blunt-ended connectors indicate inhibition, whereas arrows indicate the associated chromatin consequence. (Created with BioRender).

Epigenetic drugs encompass a diverse class of chemical modulators that target key chromatin regulators. These agents aim to reverse abnormal gene silencing or activation, offering therapeutic potential across cancer,^61^ fibrosis, and other diseases driven by epigenetic dysregulation (Figure 2; Table 3).

**Table 3:** Major classes of epigenetic drugs and their mechanisms of action

Drug class	Examples	Mechanism of action
DNMT inhibitors	azacitidine; decitabine^62^	nucleoside analogs that incorporate into DNA and trap DNMTs, leading to passive DNA demethylation
HDAC inhibitors	vorinostat; romidepsin; panobinostat; belinostat^63^	inhibit histone deacetylases, causing hyperacetylation and chromatin relaxation. reactivation of silent genes
HMT inhibitors	tazemetostat (EZH2 inhibitor)^64^	inhibit histone methyltransferases (e.g., EZH2), reducing H3K27me3 to reactivate silenced genes
BET inhibitors	JQ1 analogs (e.g., OTX015)^65^^,^^66^	block BET bromodomain proteins (e.g., BRD4) from binding acetylated histones, suppressing oncogenic transcription (e.g., MYC)

DNA methyltransferase inhibitors (DNMTi), such as 5-azacytidine and decitabine inhibit DNMT enzymes and promote passive DNA demethylation during replication.^62^ These agents are used in myelodysplastic syndromes and leukemias to reactivate silenced genes but exhibit non-specific activity, potentially inducing genomic instability.^67^ Histone deacetylase inhibitors (HDACi), including FDA-approved drugs, such as vorinostat, romidepsin, and belinostat, work by increasing histone acetylation, leading to chromatin relaxation and transcriptional reactivation.^63^ However, they also affect multiple HDAC isoforms, often resulting in systemic toxicities and a narrow therapeutic window. Emerging epi-drugs target other regulators such as HMTs and bromo extra-terminal domain (BET) proteins. EZH2 inhibitors like tazemetostat reduce H3K27me3-mediated repression and are approved for some lymphomas.^64^

Despite their potential, current epi-drugs are hampered by poor specificity, transient effects, resistance mechanisms, and suboptimal pharmacokinetics. Their broad impact on gene regulation poses safety challenges, and in many cases, their exact mechanisms are not completely understood. These limitations highlight the need for more precise and durable epigenetic therapies tailored to individual disease contexts.

### Programmable epigenetic modifiers

**Figure 3:**
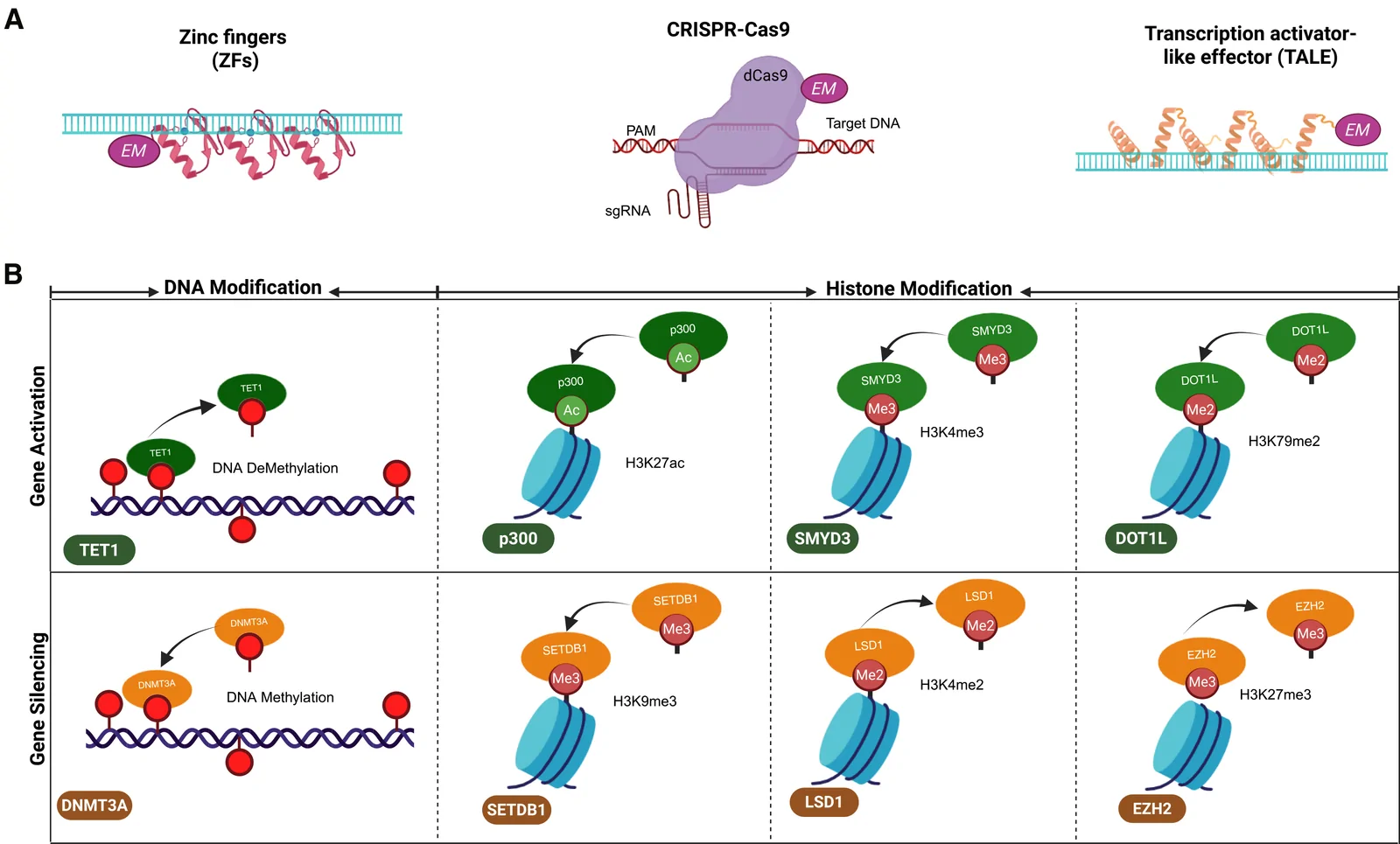
Epigenome-editing platforms and effector modules for gene regulation (A) Schematic representation of zinc finger (ZF), CRISPR-dCas9, and transcription activator-like effector (TALE) DNA-binding platforms fused to effector modules (EMs). (B) Representative effector modules associated with gene activation or silencing through targeted DNA and histone modifications, including TET1, DNMT3A, p300, SMYD3, DOT1L, KRAB, LSD1, and EZH2. (Created with BioRender).

Advances in programmable DNA-binding domains, such as ZFPs, TALEs, and catalytically inactive Cas (dCas) proteins, have enabled precise, locus-specific regulation of gene expression (Figure 3A). The most widely used DNA-binding scaffold is dead Cas9 (dCas9), a catalytically inactivated form of the *Streptococcus pyogenes* Cas9 nuclease in which point mutations in the RuvC (D10A) and HNH (H840A) endonuclease domains abolish DNA cleavage while preserving guide RNA-programmed, sequence-specific DNA binding. This allows dCas9 to act as a programmable anchor that delivers fused effector domains to defined genomic loci without introducing double-strand breaks.^68^^,^^69^ Building on these platforms, numerous synthetic and naturally derived effector domains have been developed to modulate transcriptional activity through targeted recruitment to specific genomic loci (Figure 3B) while recent innovations have further enhanced their efficiency, stability, and regulatory precision (Table 4).^68^ Throughout this review, effector sizes are given as lengths of their coding sequence (in bp). This convention directly reflects the primary bottleneck in translational applications: combining a programmable DNA-binding domain with one or more effector domains generates a single, large genetic cargo that severely constrains delivery across virtually all modalities. This constraint is most acute for adeno-associated viruses (AAVs), the most widely used *in vivo* delivery vehicle, whose strict packaging capacity of ∼4.7 kb must simultaneously accommodate regulatory elements, inverted terminal repeats, and the *S. pyogenes* dCas9 sequence (∼4.1 kb) alongside the effector payload. Consequently, large or multi-domain fusions frequently exceed this physical limit. Transient, non-viral delivery strategies shift rather than resolve this issue. For example, *in vitro*-transcribed messenger RNA (IVT mRNA) harbors constraints that scale linearly with their length, such as stability, purity and cost. Similarly, direct protein delivery entails the production of complex recombinant macromolecular complexes that is inefficient. These constraints have catalyzed intensive efforts to develop compact editors, miniature Cas orthologs, and truncated functional domains. Simultaneously, they have spurred the development of alternative vehicles, such as lipid nanoparticles (LNPs) and engineered virus-like particles (VLPs) capable of accommodating these complex RNA or protein payloads.^82^^,^^83^^,^^84^

**Table 4:** Representative programmable epigenetic-editing systems

System	Type/Function	Mechanism of recruitment or modularity	Key features/Notes
SAM (synergistic activation mediator)^70^^,^^71^	activation	RNA aptamer (MS2-MCP) system recruits p65-HSF1 activators to dCas9-VP64 complex	strong, flexible CRISPRa system; compatible with multiplex activation
CRISPR-DREAM^72^	activation	combines dCas9 with engineered transcription factors (MRTF-A, STAT1, eNRF2) via RNA scaffolds or direct fusions	compact, AAV-deliverable; suitable for robust activation of coding and noncoding genes
CRISPRon^41^	activation	dual system combining dCas9-TET1 and gRNA-bound p65-Rta domains	enables potent and heritable gene activation; reverses CRISPRoff silencing
Casilio^73^	activation	PUF-RNA-binding system attaches additional effector modules to gRNA scaffold	highly programmable; allows multiple effector recruitment combinations
Chemical epigenetic modifiers (CEMs)^74^	activation	dCas9-FKBP recruits endogenous chromatin regulators via small-molecule bridges	enables chemical control of activation with synthetic ligands
CLOuD9(CRISPR-mediated looping)^75^	chromatin looping	dual dCas9 proteins (from *S. aureus* and *S. pyogenes*) fused to ABA-inducible dimerization domains (ABI1-PYL1)	enables reversible, targeted chromatin looping to modulate gene expression and nuclear organization
SunTag^76^^,^^77^	activation or repression	tandem GCN4 peptide array on dCas9 recruits multiple scFv-fused effectors	signal amplification via multivalent effector recruitment
CRISPR-Display^78^	activation or repression	extended gRNA scaffold recruits RNAs or RNA-binding proteins to chromatin	allows lncRNA-guided recruitment of endogenous regulatory complexes
FIRE-Cas9^79^	inducible activation/repression	chemically inducible FRB/FKBP–rapamycin interaction system for effector recruitment	enables temporal control of gene regulation; reversible
CRISPRoff^41^	stable repression	multi-domain system combining KRAB and DNMT3A-3L within a single dCas9	induces durable, heritable gene silencing through DNA methylation and histone modification
Designer epigenetic modifier (DEM)^80^^,^^81^	stable repression	TALE-KRAB-DNMT3A/3L multi-domain repressor	durable, heritable gene silencing via DNA methylation and repressive histone marks

#### Programmable transcriptional repression

Repressive effectors for epigenetic editing are designed to induce chromatin compaction and transcriptional silencing. The canonical KRAB domain ∼150–300 bp, remains a widely used repressor, capable of rapid gene silencing by tethering to KAP1 and thus recruiting SETDB1, which introduces H3K9me3 and HP1 family proteins,^85^^,^^86^ though effects are often transient and may not establish durable epigenetic memory. Enhanced KRAB variants have been more recently described, such as KRAB-MECP2, which along with ensuring H3K9me3 deposition, recruits CpG-HDAC/SIN3A for more stable silencing, or the ZIM3-KRAB, which is one of the most potent silencing modules, achieving superior repression through enhanced TRIM28/KAP1 recruitment, hence achieving denser H3K9me3 deposition).^87^ HMTs such as EZH2, a core component of the polycomb repressive complex 2 (PRC2), mediate H3K27 trimethylation and can outperform KRAB in specific settings, though its large size (∼2,200 bp) complicates delivery. However, a truncated version of ∼800 bp has also been used, but EZH2 functionality is dependent on co-delivery of DNMT3L.^58^^,^^79^^,^^88^ Likewise, SUV39H1 (∼1,000 bp) is a HMTs that directly deposits the repressive H3K9me3 mark, whereas FOG1 (∼150 bp) acts as a recruitment peptide that tethers the NuRD corepressor complex, driving HDAC-mediated histone deacetylation and, indirectly, PRC2-mediated H3K27me3; the use of both in targeted epigenetic engineering remains limited. Another demethylase that has been used in direct fusions is LSD1, a large effector (∼2,500 bp) that demethylates both repressive and active histone marks. It primarily removes H3K4me2, reduces H3K27 acetylation, and may cooperate with other HDMs, broadening its impact on chromatin regulation.^15^^,^^89^ However, its impact is not only context dependent but also region specific, with markedly greater potency at enhancers than at other genomic loci. Other repressive strategies include targeted deacetylation via HDAC3 (∼1,300 bp), though its silencing efficiency is weak. Although less efficient at inducing programmable silencing, this fusion remains valuable for applications, such as dissecting the role of acetylation at specific genomic loci.^90^

Another strategy involves combining multiple effector domains in a single construct, exemplified by CRISPRoff, which combines DNMT3A-3L with KRAB (∼1,650 bp), achieving long-lasting and reversible repression through simultaneous DNA methylation and recruitment of H3K9me3 deposition, offering stability even after transient delivery.^41^ A similar system has also been reported with using TALE-based DNA-binding domains (i.e. designer epigenetic modifier, DEM) used to successfully silence *CCR5* and *CXCR4* genes simultaneously or, more recently, to silence *PDCD1* and *LAG3* to potentiate CAR T cell function.^80^^,^^81^ Alternative approaches utilize prokaryotic methyltransferases such as MQ1/M.Sss1 (∼1,200 bp), to achieve targeted *de novo* DNA methylation. While end-to-end dCas9 fusions leave a characteristic ∼20 bp unmethylated gap directly at the gRNA-binding site due to steric shielding by the dCas9 domain,^91^ split-enzyme variants (dCas9-sMTase) have been further developed to refine the spatial accuracy of the epigenetic modification.^92^ To overcome limitations of prior epigenetic silencers, Neumann et al. reported a compact, enzyme-free editor named CHARM (coupled histone tail for autoinhibition release of methyltransferase). CHARM couples an H3-tail-DNMT3L recruitment module to dCas9, TALE, or ZFP DNA binding, harnessing endogenous DNMTs to methylate promoters and establish durable, on-target transcriptional repression.^82^ The compact size of the system makes CHARM particularly attractive for delivery-constrained applications.

#### Programmable transcriptional activation

Among activating transcriptional effectors, the VP64 domain (∼150 bp) is compact, versatile, and functional across diverse cell types. It activates transcription by recruiting general transcription factors, Mediator components, and RNA polymerase II to stabilize pre-initiation complex assembly and enhance promoter accessibility. However, despite its reliability, VP64 alone often yields relatively modest activation.^93^^,^^94^ To enhance its activity, more complex constructs such as VPR, a tripartite fusion of VP64, p65, and Rta (∼1,600 bp), were developed. VPR achieves markedly stronger activation by simultaneously stabilizing pre-initiation complex assembly (VP64), recruiting Mediator and chromatin-remodeling coactivators (p65), and promoting RNA polymerase II pause release and productive elongation (Rta). However, VPR’s large size complicates delivery and has been associated with toxicity in mammalian systems.^95^^,^^96^ A more compact variant, miniVPR (∼900 bp), preserves substantial activity while reducing size constraints when combined with dCas9.^83^^,^^97^ Histone modifying enzymes further expand this tool kit. HATs such as p300 (∼1,900 bp) activate transcription by depositing H3K27ac and related acetylation marks that open chromatin, recruit bromodomain coactivators, and promote RNA polymerase II engagement at both promoters and distal enhancers.^17^^,^^85^ DNA demethylase catalytic domains such as TET1CD (∼2,200 bp) and TET3CD (∼2,800 bp) promote active DNA demethylation by oxidizing 5-methylcytosine to 5hmC, 5fC, and 5caC, which are subsequently removed through base-excision repair to restore unmodified cytosine. While both enzymes share this core dioxygenase mechanism, TET3CD is less frequently used than TET1CD.^14^^,^^98^^,^^99^^,^^100^ Their efficiency depends on methylation-sensitive promoters and their large size pose delivery hurdles. Similarly, HMTs PRDM9 (∼900 bp), DOT1L (∼1300 bp), and SMYD3 (∼1400 bp) promote transcription by catalyzing H3K4 or H3K79 methylation,^101^^,^^102^ though their activity can be weaker and highly context dependent.

Building on these enzymatic and multi-domain activators, CRISPRon employs a composite fusion of the TET1CD, p65, and Rta (∼2.9 kb) to achieve strong and coordinated gene activation when linked to dCas9. Moreover, CRISPRon can reverse CRISPRoff-induced silencing by erasing methylation marks and re-establishing an active chromatin state.^41^

#### Multicomponent recruitment platforms

Because establishment of stable epigenetic states frequently requires the coordinated action of multiple chromatin regulators, considerable effort has focused on developing multiplexed recruitment platforms capable of simultaneously recruiting several transcriptional and epigenetic effectors. These combinatorial systems exploit synergistic interactions between chromatin modifiers to achieve stronger, more durable, and more context-dependent regulation than single-effector fusions. Prominent examples include the synergistic activation mediator (SAM). A defining feature of SAM is the use of an RNA-scaffold–based recruitment strategy mediated by the MS2-MCP interaction. In this architecture, the single-guide RNA (sgRNA) is engineered to contain MS2 bacteriophage stem-loop aptamers, typically inserted into the tetraloop and stem-loop 2 regions without compromising Cas9 targeting fidelity. These RNA hairpins are specifically recognized by the MS2 coat protein (MCP), enabling stable and programmable docking of transcriptional effector domains to the sgRNA. SAM integrates dCas9-VP64 with MCP-p65-HSF1. p65 engages the mediator complex and HSF1 enhances RNA Pol II recruitment, driving robust activation.^70^^,^^71^ In contrast to SAM, the CRISPR-dCas9 recruited enhanced activation module (CRISPR-DREAM) was designed to achieve potent transcriptional activation using compact, non-viral human transactivation domains assembled through the same RNA-scaffold recruitment logic. CRISPR-DREAM employs MS2-aptamer-containing sgRNAs to recruit MCP-fused tripartite human transactivation modules, composed of domains derived from the mechanosensitive transcription factors MRTF-A, STAT1, and NRF2. Mechanistically, these domains synergize by engaging endogenous coactivators, such as p300/CBP and the mediator complex, thereby promoting chromatin accessibility and efficient RNA Pol II recruitment. By avoiding viral activation domains, CRISPR-DREAM eliminates both the severe risks of cellular toxicity, due to sequestering of endogenous transcription complexes away from normal cellular operations,^103^ and the adverse host immune clearance, due to pre-existing immunity to common viral pathogens.^104^ This architecture enables robust and specific activation across promoters, enhancers, and noncoding regulatory elements, and performs comparably to or better than SAM and VPR across multiple mammalian cell types. Importantly, the modular recruitment strategy allows CRISPR-DREAM to remain compact, portable across diverse CRISPR systems (including dCas12a and type I cascade), and to be amenable to multiplexed gene activation, addressing key delivery and scalability limitations of earlier CRISPRa platforms.^72^

Extending modular recruitment strategies beyond protein- and RNA-based scaffolds, chemical epigenetic modifiers (CEMs) introduce a chemically inducible approach to locus-specific gene activation. CEMs act as molecular bridges that simultaneously bind FKBP and endogenous chromatin regulators, most prominently BET bromodomain proteins such as BRD4, thereby concentrating native transcriptional coactivators at the targeted locus. Mechanistically, BRD4 recruitment promotes local histone acetylation, enhancer activation, and RNA Pol II engagement, leading to robust transcriptional induction. Notably, the use of a biorthogonal bump-and-hole FKBP(F36V)-ligand pair enables highly specific, dose-dependent, and reversible gene activation, as demonstrated by BRD4 ChIP-seq showing precise enrichment restricted to sgRNA-defined target loci with minimal genome-wide redistribution. This chemically tunable architecture provides precise temporal control and scalability advantages over permanent fusion-based activators, while leveraging endogenous epigenetic machinery rather than synthetic transcriptional domains.^105^

Continuing the theme of modular RNA-guided recruitment, the Casilio system introduces a programmable RNA-protein tethering strategy based on Pumilio/FBF (PUF) RNA-binding domains. In this architecture, sgRNAs are extended with short, linear PUF-binding sequences that serve as customizable docking sites for PUF-effector fusion proteins, enabling recruitment without perturbing Cas9 targeting. A key mechanistic advantage of Casilio is its capacity for multivalent effector loading, as multiple PUF-binding sites can be appended to a single sgRNA to locally concentrate several copies of an effector at the targeted locus, thereby amplifying regulatory output. Furthermore, the use of engineered PUF variants with distinct RNA specificities enables orthogonal and combinatorial recruitment of different effectors, supporting simultaneous activation, repression, or chromatin remodeling at selected genomic sites. This flexible and scalable design allows Casilio to function as a versatile platform for transcriptional regulation, epigenetic modification, and locus-specific signal amplification, although at the cost of increased optimization requirements for sgRNA architecture and effector stoichiometry.^73^

In addition to RNA-dependent recruitment platforms, peptide-based systems have been developed for site-specific effector recruitment. The SunTag system employs tandem repeats of the GCN4 epitope (yeast transcription factor) to recruit multiple copies of effectors, such as p300, DNMT3A/3L, or EZH2-PRC2, which are fused to a single-chain variable fragment (scFv) antibody that specifically binds to GCN4, thereby amplifying transcriptional or repressive outputs beyond what fusions can achieve.^77^^,^^106^ While this split system significantly enhances efficacy through multivalent effector recruitment, a persistent challenge remains: the anti-GCN4 scFv is itself a sizable module (∼750 bp) that, when paired with an effector domain, imposes a strict delivery constraint. A promising alternative is the self-assembling coiled-coil (CC) platform. Based on orthogonal CC modules encoded by sequences of only ∼84 bp, this compact system enables highly modular, programmable effector recruitment. By assembling specific CC heterodimers, this system creates protein origami-like scaffolds that can fold into nanostructures, offering precise stoichiometric control and the potential for *in vivo* biocompatibility. This platform can also serve as an alternative to SunTag, as the CC domain fused to the DNA-binding domain can be multimerized, similar to GCN4 repeats, to achieve comparable levels of effector recruitment and signal amplification.^107^^,^^108^ Although these systems have proven effective, they have been used predominantly for gene activation. However, their split and modular nature suggests that they could be equally well suited for targeted gene repression. Collectively, these modular recruitment platforms illustrate the transition from single-effector editors toward combinatorial epigenetic engineering strategies that simultaneously engage multiple regulatory pathways to achieve synergistic and durable transcriptional control.

### Machine learning for epigenetic editor design

A rapidly growing direction in epigenetic engineering is the application of artificial intelligence and machine learning to support the rational design of programmable epigenetic editors by predicting editing outcomes from DNA sequence and target-specific chromatin features. Because editing efficacy is strongly influenced by the local epigenetic landscape, machine learning models that integrate chromatin accessibility, DNA methylation, histone modifications, and transcriptional activity are particularly well suited to guide both target selection and effector choice. Rather than relying solely on sequence information, these models incorporate the endogenous regulatory state of the target locus, thereby enabling context-aware predictions that more closely reflect biological editing outcomes. In this direction, Song and colleagues, for example, trained machine learning models on integrated epigenomic and transcriptomic datasets across multiple ENCODE cell types to predict gene expression from histone post-translational modifications, and subsequently used these models to anticipate the transcriptional consequences targeted H3K27ac deposited by dCas9-p300.^109^ This study illustrates how predictive modeling can directly inform epigenome editing by linking pre-existing chromatin features to the expected outcome of a specific editing strategy. More broadly, deep-learning models trained on large CRISPR activity datasets now outperform traditional rule-based approaches for predicting on-target efficiency. Importantly, their predicted accuracy improves substantially when chromatin accessibility and other epigenetic features are incorporated as input variables, highlighting that editor performance is determined not only by guide sequence but also by the surrounding chromatin environment.^110^ Recent transfer-learning approaches further refine these predictions for individual cell types by adapting pretrained models to cell-specific epigenomic datasets, enabling increasingly accurate, context-dependent prediction of editing efficiency.^111^ As comprehensive epigenomic datasets continue to expand, AI-driven frameworks are expected to evolve from guide RNA-prediction tools into integrated decision-support systems capable of recommending target loci, selecting optimal effector domains, and predicting the durability of installed epigenetic states based on the endogenous chromatin landscape. Such approaches closely align with the central concept of this review: that characterization of the target epigenetic state provides the foundation for the rational design of programmable epigenetic editing strategies.

### Epigenetic memory and the heritability of installed marks

Beyond installing a mark efficiently, a defining requirement for therapeutic epigenetic editing is whether that mark persists once the editor is withdrawn. This durability, often termed epigenetic memory, depends less on the engineered effector itself than on its ability to engage the cell’s endogenous epigenetic maintenance machinery, because a deposited modification is only heritable if it can be faithfully propagated through DNA replication and cell division. For DNA methylation, this process is mediated primarily by DNMT1, which recognizes hemimethylated CpG sites generated during semiconservative DNA replication and, together with its cofactor UHRF1, restores the parental methylation pattern on the newly synthetized strand, thereby propagating the epigenetic mark across cell generations.^112^^,^^113^ Maintenance of transcriptionally repressive chromatin is further reinforced through crosstalk with histone modifications: H3K9me3 is read by both the RFTS domain of DNMT1 and the tandem Tudor domain of UHRF1,^112^^,^^114^ while HP1 proteins bind H3K9me3 to promote heterochromatin spreading and stabilization.^115^ These interconnected maintenance pathways explain why combinatorial editors such as CRISPRoff or DEMs, which simultaneously deposit DNA methylation and H3K9me3, often achieve repression that persists after transient editor delivery, whereas effectors that recruit a single repressive pathway (for example, KRAB-mediated H3K9me3 in the absence of DNA methylation) often exhibit progressive loss of repression once the editor is removed.^41^^,^^80^ Importantly, these findings indicate that durable epigenetic editing is achieved not simply by depositing a regulatory mark but by initiating endogenous maintenance circuits capable of perpetuating that state. Consequently, rational editor design should consider not only which epigenetic mark is required to modulate transcription but also whether the selected effector, or combination of effectors, is able to recruit or engage the endogenous maintenance machinery necessary for stable inheritance of the edited state at the target locus.

## Therapeutic applications

### Cancer epigenetics and therapy

Cancer is the field where epigenetic therapies are most advanced. Multiple epigenetic drugs, such as the DNMTi and HDACi mentioned previously, are FDA-approved for hematologic malignancies, and numerous clinical trials are evaluating new agents and combination regimens. Tumors often exhibit a CpG island methylator phenotype (CIMP), characterized by widespread promoter hypermethylation and silencing of tumor suppressor genes, along with global changes in histone modification and chromatin organization. Epigenetic therapy in cancer aims to reprogram these aberrant patterns. Combination approaches using multiple epigenetic drugs or pairing epigenetic drugs with conventional chemotherapy or immunotherapy have shown promising results in trials. For example, therapeutically derepresses transposable elements (TEs) in cancer cells. The resulting accumulation of TE-derived transcripts triggers innate antiviral pathways that mimic a viral infection response, thereby transforming immunologically “cold” tumors into “hot” tumors through interferon production and viral-like antigen presentation.^116^ In response to this inflammatory cascade, tumor cells adaptively upregulate immune checkpoint genes to escape recognition; paradoxically, this protective mechanism renders the tumor sensitive to immune checkpoint inhibition.^62^^,^^67^^,^^116^ Another successful cancer therapy involves the use of T cells engineered to express chimeric antigen receptors (CARs). However, also in this context T cell exhaustion remains a major challenge. Exhaustion is a progressive state of dysfunction induced by chronic antigen exposure, where T cells lose their proliferation and cytokine-production capabilities due to sustained upregulation of inhibitory receptors like PD-1 and LAG3.^117^ Multiplexed epigenetic silencing of the corresponding inhibitory-receptor genes can prevent this dysfunctional transition, thereby preserving CAR T cell effector function and anti-tumor durability.^118^ Notably, Azcona et al. have shown that multiplexed epigenetic silencing of PD-1 and LAG3 encoding genes in CAR T cells led to durable gene silencing with preserved T cell function. This epigenetic approach offers a crucial safety advantage over conventional CRISPR-knockout methods: by bypassing the need for DNA double-strand breaks (DSBs), it entirely eliminates the risks of genotoxicity, chromosomal translocations, and deleterious indels that typically occur when multiple genomic loci are targeted simultaneously.^81^ Similarly, by reversing epigenetic silencing of critical genes, these treatments can drive cancer cells into more differentiated, less aggressive states or enhance their susceptibility to immune attack.^119^

### Inherited disorders

Many neurodevelopmental and neurodegenerative disorders have an epigenetic component to their pathology. In conditions, such as Rett syndrome, Fragile X syndrome, and Alzheimer’s disease, characteristic aberrations in DNA methylation or histone acetylation have been observed. Pioneering work by Segal and colleagues demonstrated that the silenced paternal *UBE3A* allele in Angelman syndrome can be restored not by activating *UBE3A* directly, but by using an engineered transcriptional repressor to silence the *UBE3A* antisense transcript (*UBE3A-ATS*) that keeps it inactive, thereby reactivating endogenous paternal *UBE3A* brain-wide in a mouse model.^120^ Similarly, for another imprinting disorder, Prader-Willi syndrome (PWS), researchers have recently shown the activation of the normally silenced maternal gene cluster in PWS using dCas9-TET1 to demethylate the imprinting control region in patient-derived cells.^121^ Epigenetic editing also holds considerable promise for other inherited diseases. In β-hemoglobinopathies like sickle cell disease and β-thalassemia, a key therapeutic strategy is to induce fetal hemoglobin (HbF) production by silencing the developmental repressor BCL11A in adult erythroid cells. Traditional gene-editing approaches^122^ achieve this by disrupting the *BCL11A* erythroid enhancer or editing the *HBG1/2* promoters, but epigenetic editors can accomplish a similar outcome without cutting DNA.^123^ For example, targeted CpG demethylation at the γ-globin (HBG) promoters, either alone or combined with H3K27 acetylation through TET1-dCas9 and dCas9-CBP epigenome editors, has been shown to increase fetal hemoglobin expression to therapeutically relevant levels.,^124^^,^^125^ Unlike traditional gene therapy, which may risk overexpression or random integration, epigenetic editing can selectively silence a disease-causing allele or activate a silenced beneficial allele while preserving the normal counterpart.

### Infectious diseases

Chronic viral infections may also be tackled with epigenetic editing strategies. A notable example is hepatitis B virus (HBV), which persists in the nucleus of infected hepatocytes as a covalently closed circular DNA (cccDNA) mini-chromosome that is difficult to eradicate. Epigenetic therapy is being applied to silence this viral DNA: an mRNA-based lipid nanoparticle delivery of a CRISPR-dCas9-KRAB repressor system targeting HBV is now in clinical trial as the first epigenome-editing therapy in humans.^126^ This experimental treatment (EPI-003) aims to deposit repressive heterochromatin marks on the HBV mini-chromosome and permanently shut down viral gene expression, offering a functional cure without altering host DNA.^127^ Similar epigenetic approaches are under exploration for HIV, i.e., simultaneous silencing of *CCR5* and *CXCR4,* serve as essential entry receptors for R5- and X4-tropic HIV strains, respectively, have been achieved using DEM, demonstrating mutation-free therapeutic approach.^80^

## Conclusions

Advances in epigenetic editing have opened new possibilities for precise, reversible, and targeted regulation of gene expression. Traditional epigenetic drugs, while clinically useful, affect the genome broadly and often lead to off-target effects and toxicity. In contrast, programmable platforms, such as ZFPs, TALEs, and CRISPR-based systems enable locus-specific modifications without altering the underlying DNA sequence. However, epigenetic editing should be preceded by a detailed understanding of target locus by analyzing the epigenetic profile. These insights allow researchers to choose the most suitable effector domains (e.g., methyltransferases, acetyltransferases, repressors, and activators) based on the local chromatin environment, increasing both the precision and effectiveness of the intervention.

Clinical applications of programmable epigenetic editing are being explored in cancer, neurological disorders, imprinting syndromes and other inherited disorders, and chronic viral infections. These tools can reactivate silenced tumor suppressors, silence disease-causing alleles, or target latent viral genomes—all without introducing permanent genetic changes. This reversibility makes epigenetic editing a safer and more adaptable alternative to conventional gene therapy. Despite progress, several challenges remain. Despite substantial advances, several challenges must be addressed before widespread clinical implementation. Efficient and tissue-specific delivery of epigenetic editors and long-term maintenance of therapeutic effects remain major hurdles. Although programmable epigenetic editors generally exhibit lower off-target activity than conventional nuclease-based genome-editing systems, unintended deposition or removal of epigenetic marks at partially matched genomic sites is a possibility. In addition, the spreading of chromatin modifications beyond the intended target locus may influence neighboring regulatory elements and gene expression. Continued improvements in editor design, targeting specificity, and comprehensive safety assessments are essential for successful clinical translation. Overall, the integration of high-resolution epigenetic profiling with programmable epigenetic editing provides a promising foundation for precision medicine, enabling tailored regulation of gene expression to achieve durable therapeutic outcomes.

## Acknowledgments

We acknowledge funding from the 10.13039/501100001659German Research Foundation (10.13039/501100001659DFG, grant MU 3861/5-1) to C.M. A.F. is a recipient of a fellowship from the 10.13039/100021828German Academic Exchange Service (10.13039/501100001655DAAD) and Higher Education Commission (HEC) Pakistan. We would like to thank all the members of the ITG R&D department for productive discussions. The article processing charge was funded by the University of Freiburg’s Open Access Publishing funding program.

## Author contributions

Conceptualization, A.F., S.H., and C.M.; writing – original draft preparation, A.F.; writing – review and editing, S.H. and C.M.; visualization, A.F.; project administration, C.M.; funding acquisition, C.M. All authors have read and agreed to the published version of the manuscript.

## Declaration of interests

C.M. is an advisor to Lepton Pharmaceuticals, uniQure biopharma, and CRISP-HR Therapeutics.

## Declaration of generative AI and AI-assisted technologies in the writing process

We acknowledge the use of ChatGPT to improve clarity, readability, and grammatical accuracy of the manuscript text and journal-specific formatting. The scientific content, conceptual framework, and overall design of the review were independently developed and executed by the authors.
